# Vascular Complications in Patients with Hepatocellular Carcinoma Treated with Sorafenib

**DOI:** 10.3390/cancers12102961

**Published:** 2020-10-13

**Authors:** Katharina Pomej, Bernhard Scheiner, Dabin Park, David Bauer, Lorenz Balcar, Tobias Meischl, Mattias Mandorfer, Thomas Reiberger, Christian Müller, Michael Trauner, Matthias Pinter

**Affiliations:** 1 Department of Internal Medicine III, Division of Gastroenterology and Hepatology, Medical University of Vienna, 1090 Vienna, Austria; katharina.pomej@meduniwien.ac.at (K.P.); bernhard.scheiner@meduniwien.ac.at (B.S.); n1442022@students.meduniwien.ac.at (D.P.); david.bauer@meduniwien.ac.at (D.B.); n1623284@students.meduniwien.ac.at (L.B.); tobias.meischl@meduniwien.ac.at (T.M.); mattias.mandorfer@meduniwien.ac.at (M.M.); thomas.reiberger@meduniwien.ac.at (T.R.); christian.j.mueller@meduniwien.ac.at (C.M.); michael.trauner@meduniwien.ac.at (M.T.); 2 Liver Cancer (HCC) Study Group Vienna, Medical University of Vienna, 1090 Vienna, Austria; 3 Vienna Hepatic Hemodynamic Laboratory, Medical University of Vienna, 1090 Vienna, Austria; 4 Rare Liver Disease (RALID) Centre of the ERN RARE-LIVER, Medical University of Vienna, 1090 Vienna, Austria; 5 Christian Doppler Laboratory for Portal Hypertension and Liver Fibrosis, Medical University of Vienna, 1090 Vienna, Austria

**Keywords:** hepatocellular carcinoma, sorafenib, vascular complications, thromboembolic events, bleeding complications, metabolic risk factors, cardiovascular risk

## Abstract

**Simple Summary:**

Inhibition of vascular endothelial growth factor receptor (VEGFR) signaling is associated with an increased risk of thromboembolic and bleeding complications in cancer patients and may cause fatal side effects. The multityrosine kinase inhibitor sorafenib represents an important treatment option for patients with hepatocellular carcinoma (HCC), as all currently approved second-line treatments have only been approved in sorafenib-experienced patients. However, safety concerns regarding sorafenib treatment in patients with cardiovascular disease have been raised. Therefore, we retrospectively analyzed the incidence of arterial/venous thromboembolic and bleeding complications in 252 patients with HCC treated with sorafenib. Importantly, the incidence of arterial/venous thromboembolic events was low even though more than half of patients had advanced liver dysfunction and a substantial cardiovascular risk according to Framingham risk score. Bleeding complications occurred in every fifth patient. In conclusion, sorafenib represents a safe treatment option even in patients with an increased cardiovascular risk.

**Abstract:**

VEGF(R)-targeted therapies are associated with an increased risk of thromboembolism and bleeding, which might be pronounced in patients with increased cardiovascular risk. Nevertheless, sorafenib represents an important treatment option in patients with hepatocellular carcinoma (HCC). We retrospectively investigated the risk of arterial/venous thromboembolic and bleeding events in 252 patients treated with sorafenib for HCC between 05/2006 and 03/2020 at the Medical University of Vienna. Cardiovascular risk was assessed using Framingham score. Eight patients (3.2%) experienced 11 arterial/venous thromboembolic events. Only two patients (0.8%) developed arterial thromboembolism even though cardiovascular risk was low, intermediate, and high in 15 (8.7%), 104 (60%), and 54 (31.2%) of 173 assessable patients. Median overall survival (OS) was shorter in the high risk vs. low/intermediate risk group 7.4 (95% CI: 3.4–11.3) vs. 10.0 (95% CI: 6.8–13.2 months) and independently associated with OS in multivariable analysis HR: 1.53 (95% CI: 1.07–2.19; *p* = 0.019). Forty-eight (19%) patients experienced a bleeding, most commonly gastrointestinal bleeding (14%) followed by epistaxis (4.7%). Advanced liver dysfunction was not associated with an increased incidence of bleeding/venous thromboembolism. Sorafenib represents a safe treatment option even in patients with increased cardiovascular risk. Bleeding complications were comparable with previous reports, even though patients with more advanced liver disease were included.

## 1. Introduction

Hepatocellular carcinoma (HCC) accounts for 90% of primary liver cancers and represents the second most common cause of cancer-related death in men and the sixth leading cause of cancer-related death in women worldwide [1,2]. Most HCC cases (80–90%) develop in patients with underlying liver cirrhosis [3,4]. Despite clear screening recommendations [2], HCC is still often detected in advanced stages, where palliative treatment with systemic therapy is the only available option [5].

Since its approval in 2007, the multi-tyrosine kinase inhibitor (TKI) sorafenib remained the standard of care in patients with advanced HCC for over a decade. Only recently, lenvatinib was shown non-inferior to sorafenib [6], and even more importantly, the combination of atezolizumab plus bevacizumab improved both co-primary endpoints overall survival (OS) and progression-free survival (PFS) compared to sorafenib, and thus will become the new frontline treatment [7,8,9]. Nevertheless, sorafenib will still play a crucial role in the treatment algorithm of HCC, as all currently approved second-line treatments (regorafenib, cabozantinib, ramucirumab) have been approved in sorafenib-experienced patients [5,10,11,12,13].

Sorafenib blocks vascular endothelial growth factor receptors (VEGFRs) among several other signaling pathways involved in angiogenesis and tumor growth [14]. VEGF(R)-targeting agents are associated with an increased risk of thromboembolic events, and particularly arterial complications account for a significant proportion of fatal events in cancer patients receiving anti-VEGF(R) treatments [15,16,17]. Mechanistically, blocking VEGF signaling induces hypertension as well as endothelial dysfunction which promotes vasospasm/vasoconstriction, atherosclerosis, activation of platelets, hemostatic imbalance, and vascular thrombosis [17]. An increased risk of ischemic and cardiovascular events was observed during sorafenib treatment [18,19].

Hemorrhage is another well-known and typical complication of VEGF(R)-targeted agents [20] and a major concern in patients with underlying liver cirrhosis. In the pivotal phase III SHARP trial, the incidence of any-grade hemorrhage was numerically increased in sorafenib-treated HCC patients (7% vs. 4%), but not that of higher grade bleeding events [19]. Notably, only patients with well-preserved liver function (Child–Pugh class A) were included [19], and the bleeding risk may be higher in patients with more advanced liver function impairment.

The aim of this study was to evaluate the incidence of bleeding events as well as arterial and venous thromboembolic complications in patients with hepatocellular carcinoma treated with sorafenib in a large, unselected real-world cohort. Particularly, we focused on investigating the incidence of ischemic complications with respect to the underlying cardiovascular risk, and on assessing the incidence of bleeding complications with respect to underlying liver dysfunction.

## 2. Results

### 2.1. Patients

**Figure 1:**
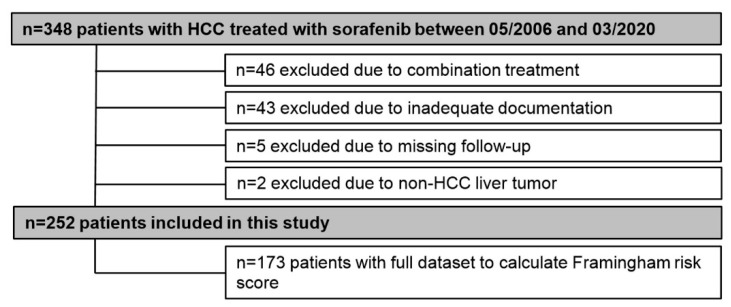
Patient flow chart.

In total, 348 patients were treated with sorafenib between 05/2006 and 03/2020 at the Division of Gastroenterology & Hepatology, Medical University of Vienna. Of those, 96 patients were excluded from this study for the following reasons: Combination therapy (*n* = 46), non-HCC tumor (*n* = 2), inadequate documentation (*n* = 43), and insufficient follow-up (*n* = 5) (Figure 1).

**Figure 2:**
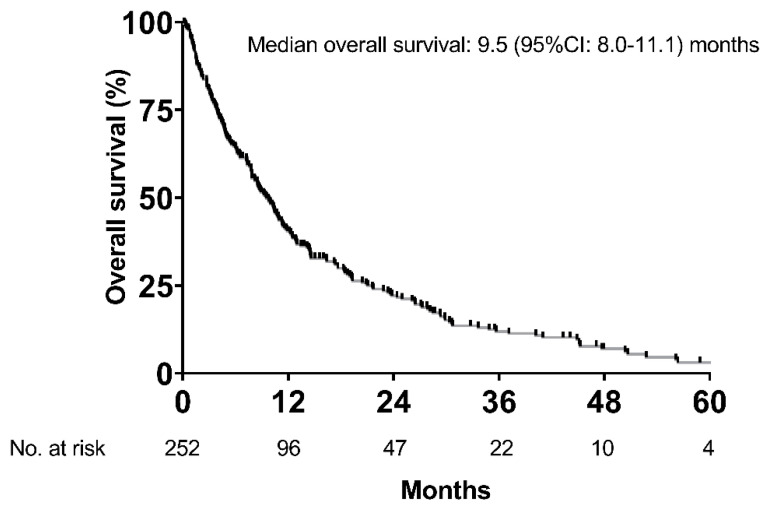
Overall survival of the whole cohort.

Consequently, 252 patients were included for final analyses, and detailed patient characteristics are shown in Table 1. The majority of patients was male (*n* = 215, 85%) with a mean age of 66 ± 9.4 years. Alcoholic liver disease (37%) and viral hepatitis (28%) were the main etiologies of liver cirrhosis. One-hundred and fourteen (45%) patients had Child–Pugh stage A, while 95 (38%) and 43 (17%) patients had Child–Pugh stage B and C, respectively. The majority of patients (58%) had advanced stage HCC as indicated by BCLC C (*n* = 146, 58%). More than half (59%) of patients did not present with tumor-related symptoms (ECOG-PS 0). Median time of sorafenib treatment was 4.3 (95% CI: 3.3–5.3) months. Median OS of the entire cohort was 9.5 (95% CI: 8.0–11.1) months (Figure 2).

### 2.2. Prevalence of Cardiovascular Risk Factors

Well-known cardiovascular risk factors were quite common in our cohort (Table 1). While 95 patients (38%) had diabetes mellitus, 58 patients (23%) were obese, 154 (62%) had arterial hypertension, and 106 (42%) were smokers. Additionally, hypercholesterinemia was present in every fourth patient (*n* = 55, 24%) and 15% of patients (*n* = 38) were receiving statin treatment, while hypertriglyceridemia was found in 16 patients (7%). At sorafenib initiation, a history of coronary heart disease was known in 23 patients (9%), while arterial occlusive disease was knowingly present in 22 patients (8%).

### 2.3. Arterial and Venous Thromboembolic Complications

In total, eight patients (3.2%) developed 11 thromboembolic events during treatment with sorafenib (Table 2). One patient (<1%) developed a myocardial infarction (MCI) and one patient (<1%) presented with an acute ischemic stroke. Additionally, four patients were diagnosed with deep vein thrombosis (DVT, 2%) and two patients experienced pulmonary embolism (PE). New occurrence of portal vein thrombosis was observed in three patients (1%). The prevalence of well-known risk factors for the development of thromboembolic events (i.e., smoking, hypertriglyceridemia) was proportionally higher in patients developing these events during sorafenib treatment as demonstrated in Supplementary Materials (Table S1).

The cumulative incidence rate for thromboembolic complications (including both arterial and venous events) was 2.42% at 12 months, while it was 0.8% for arterial thromboembolic events and 2.4% for venous events at 12 months. According to a competing risk analysis comparing the cumulative incidence of thromboembolic events between patients with Child–Pugh class A vs. B/C considering death as a competing risk, the cumulative incidence of thromboembolic events at six months (Child–Pugh A vs. B/C: 2.7% vs. 1.5%) as well as at 12 months (Child–Pugh A vs. B/C: 3.6% vs. 1.5) was comparable between both groups (adjusted subdistribution hazard ratio (aSHR: 0.27 (95% CI: 0.05–1.35); *p* = 0.11) Supplementary Materials (Figure S1).

**Figure 3:**
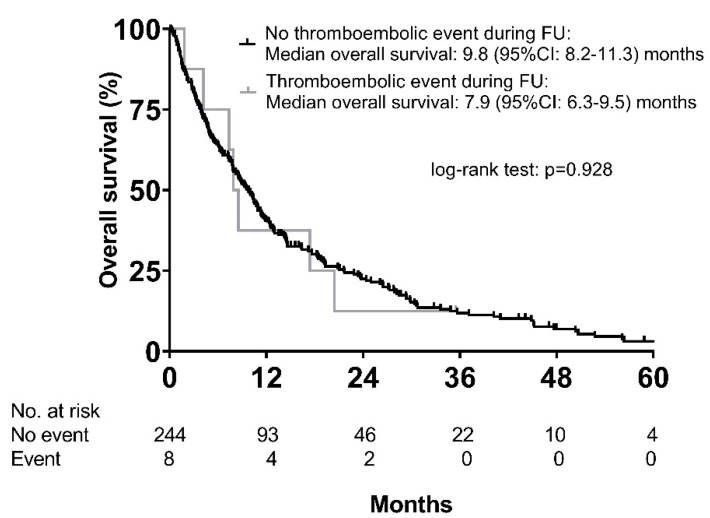
Overall survival according to the occurrence of thromboembolic events.

Almost all patients with thromboembolic events had multifocal hepatocellular carcinoma (88%) with macrovascular invasion (75%). The median time from the start of sorafenib treatment to the event was 3.8 (95% CI, 3.1–4.5) months. As a result of the event, two patients (25%) died within 14 days, while sorafenib treatment was stopped in two (25%), and an additional four patients (50%) received anticoagulation. Median OS was not significantly different between patients with and without a thromboembolic event (7.9 (95% CI: 6.3–9.5) months vs. 9.8 (95% CI: 8.2–11.3) months; *p* = 0.928) (Figure 3), even after analyzing only Child–Pugh A patients (8.5 (95% CI: 0.0–19.9) months vs. 13.0 (95% CI: 10.6–15.3) months; *p* = 0.768).

### 2.4. Incidence of Arterial Vascular Complications and Impact of Cardiovascular Risk on Survival

Only two out of 252 patients (0.8%) developed arterial ischemic complications during sorafenib treatment, reflecting a cumulative incidence rate of 0.8% at 12 months. Cardiovascular risk was assessed by means of the Framingham risk score in those patients where all variables required to calculate the Framingham score were available (*n* = 173). Fifteen patients (8.7%) had a low risk for cardiovascular events (<10%), 104 patients (60%) were classified as having an intermediate risk (10–20%), and 54 patients (31.2%) had a high-risk (>20%) for CVD events within 10 years.

The patient developing MCI had a Framingham risk score of 11 points resulting in a risk of 7.3% for the development of cardiovascular events within 10 years. The patient who experienced an ischemic stroke achieved 17 points, corresponding to a high risk of 29.4%. Sorafenib dose at the time of event was 800 mg for the MCI and 200 mg for the stroke, respectively. As a result of the event, sorafenib treatment was interrupted in one (stroke) and discontinued in the other patient (MCI). Despite undergoing percutaneous coronary intervention (PCI), the MCI was fatal.

Finally, we assessed time to progression (TTP) and OS according to cardiovascular risk (Framingham risk score). Due to the low number of patients with low cardiovascular risk, patients with low and intermediate risk were grouped for analyses. Median TTP was significantly different between patients with different Framingham risk groups, as it was 6.5 (95% CI: 4.9–8.1) months in the low/intermediate group (*n* = 119) and 3.8 (95% CI: 2.4–5.2) months (*p* = 0.019) in the high-risk group (*n* = 54) (Figure S2).

**Figure 4:**
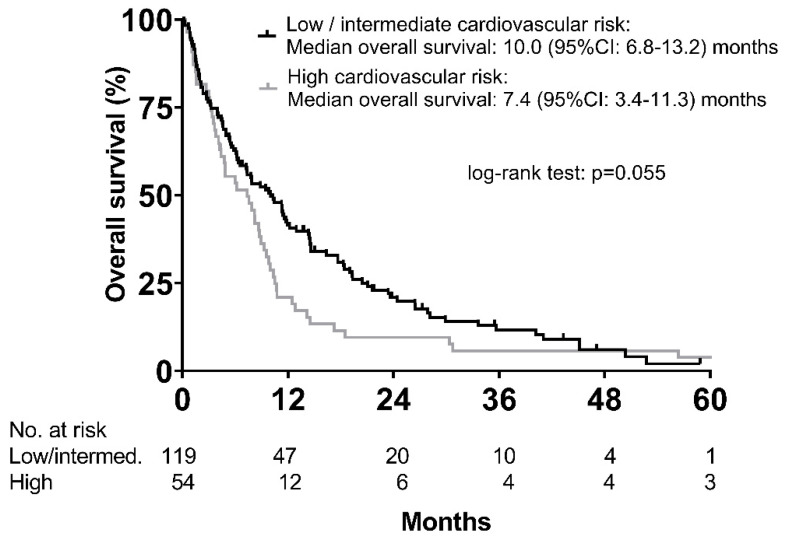
Overall survival according to cardiovascular risk categories as defined by Framingham risk score.

Median overall survival was shorter in the high risk (*n* = 54) vs. the low/intermediate risk (*n* = 119) groups (7.4 (95% CI: 3.4–11.3) months vs. 10.0 (95% CI: 6.8–13.2) months; *p* = 0.055) (Figure 4). Other variables associated with OS in univariable analysis are shown in Supplementary Materials Table S2.

In multivariable Cox regression analysis (Table 3), Framingham risk score (HR 1.53 (95% CI: 1.07–2.19); *p* = 0.019) was significantly associated with OS independently of tumor stage (BCLC B: HR: 1.04 (95% CI: 0.48–2.24), *p* = 0.928; BCLC C: HR: 1.21 (95% CI: 0.63–2.30), *p* = 0.568; BCLC D: HR: 2.34 (95% CI: 1.16–4.71), *p* = 0.018) and AFP levels (AFP > 400 IU/mL: HR: 2.04 (95% CI: 1.44–2.90); *p* < 0.001).

### 2.5. Bleeding Complications

In total, 48 (19%) patients experienced a bleeding complication, reflecting a cumulative incidence rate of 17.6% at 12 months. The most common events were gastrointestinal bleeding (*n* = 35, 14%) followed by epistaxis (*n* = 12, 4%). Less common events included intracerebral hemorrhage, hemorrhoidal hemorrhage, and gingival bleeding (one patient each, <1%). Seventeen (6.7%) patients had a bleeding complication grade 3 or higher, including three fatal (grade 5) events (*n* = 2, gastrointestinal bleeding; *n* = 1 intracerebral hemorrhage).

In order to determine potential patient characteristics associated with the development of bleeding, baseline characteristics were compared between patients with vs. without a bleeding event, but no significant association was found (Table S3). Importantly, liver function, as indicated by Child–Pugh stage, did neither impact on the incidence of gastrointestinal bleeding (Child–Pugh A vs. B vs. C: *n* = 13 (11.4%) vs. *n* = 13 (13.7%) vs. 9 (21%); *p* = 0.305) nor on the incidence of epistaxis (Child–Pugh A vs. B vs. C: *n* = 5 (4.4%) vs. *n* = 6 (6.3%) vs. *n* = 1 (2.3%); *p* = 0.576) (Table 4). The occurrence of any bleeding event was also not different between Child–Pugh stages (Child–Pugh A vs. B vs. C: *n* = 20 (17.5%) vs. *n* = 18 (18.9%) vs. *n* = 10 (23.3%); *p* = 0.718).

**Figure 5:**
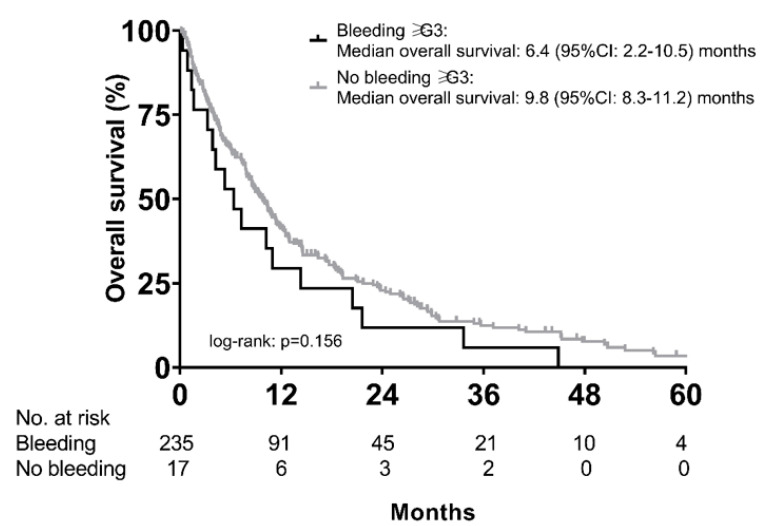
Overall survival of patients with vs. without high-grade (grade ≥ G3) bleeding events during sorafenib treatment.

Median OS of patients with (*n* = 48) and without (*n* = 204) any bleeding event was not significantly different (9.3 (95% CI: 6.7–11.9) months vs. 9.8 (95% CI: 7.7–11.8) months; *p* = 0.854). Similarly, there was only a numerical difference in median OS between patients with (*n* = 17) vs. without (*n* = 31) high grade (grade ≥ 3) bleeding (6.4 (95% CI: 2.2–10.5) months vs. 9.8 (95% CI: 8.3–11.2) months, *p* = 0.156) (Figure 5).

### 2.6. Incidence of Vascular (Thromboembolic and Bleeding) Events According to Sorafenib Starting Dose

In order to evaluate the risk of vascular complications with respect to sorafenib starting dose, we performed a competing risk analysis (death as competing risk). While most patients (*n* = 181; 72%) were started with the full dose of 800 mg daily, 60 patients (24%) were started with 400 mg and only one patient with 200 mg (>1%). In 10 patients (4%), the exact starting dose was unknown. We grouped patients with 200 mg and 400 mg starting dose to a “reduced dose” group and compared the incidence of vascular events to patients who were initiated on the full dose. The incidence of vascular events was comparable between patients who started with the full vs. the reduced sorafenib dose (cumulative incidence at six months: reduced dose vs. full dose: 21.3% vs. 14.5%; at 12 months: reduced dose vs. full dose: 23.0% vs. 17.9%; adjusted subdistribution hazard ratio (aSHR: 0.83 (95% CI: 0.44–1.56), *p* = 0.56) (Supplementary Materials Figure S3).

## 3. Discussion

Arterial ischemic complications are rare but well-recognized adverse events of VEGF(R)-targeted therapies [17]. The incidence of cardiac ischemia/infarction was significantly higher in renal cell carcinoma (RCC) patients treated with sorafenib when compared to placebo (4.9% vs. 0.4%; *p* = 0.01) [18]. An increased number was also found in a phase III trial (SHARP) in patients with HCC (3% vs. 1%), even though patients with unstable coronary heart disease or recent myocardial infarction were excluded from participation [19].

Only little is known about the risk of arterial vascular complications with respect to the cardiovascular risk profile in patients treated with anti-VEGF(R) therapies. In our real-world cohort of HCC patients receiving sorafenib, only two patients (0.8%) experienced arterial ischemic complications, corresponding to a cumulative incidence rate of 0.8% at 12 months. This is of particular interest, since we included a considerable number of patients with a high risk for developing cardiovascular events as according to Framingham risk score, and 9% and 8% of the cohort had a history of coronary heart disease and arterial occlusive disease, respectively. Thus, our data suggest that sorafenib can be safely used in patients with risk factors for the development of cardiovascular events, and even in those with a high cardiovascular risk. Some cardiovascular risk factors, including obesity, diabetes, or metabolic syndrome, are associated with an increased risk for HCC development [21]. Thus, they are frequently found in patients with HCC, especially in those with non-alcoholic fatty liver disease (NAFLD), which is a leading underlying etiology in HCC patients and on the rise globally [22,23]. In our cohort, 62% of patients were diagnosed with arterial hypertension, almost half of patients were smoking (42%) and 38% had diabetes mellitus. Even though we did not observe a high rate of cardiovascular events in our patients, individuals with a high cardiovascular risk had a shorter overall survival when compared to those with low or intermediate risk, and cardiovascular risk was independently associated with OS after correcting for tumor stage and AFP. This highlights the importance of an optimal management and medical treatment of cardiovascular risk factors [24]—even in patients with advanced malignancies with a dismal prognosis.

Patients with cancer have an increased risk for developing venous thromboembolism. The incidence rates vary between 1% to 20%, depending on various risk factors, including tumor type [25,26]. Additionally, patients with advanced liver cirrhosis are at higher risk of developing bleeding as well as venous thromboembolic complications, as they have a more fragile hemostatic balance [27,28,29]. As most patients with HCC suffer from underlying cirrhosis [4], bleeding and venous thromboembolism are of major interest in HCC patients treated with anti-VEGF(R) treatments, as VTE is not only a complication of malignancy but may also develop as a consequence to treatment with different VEGF(R) inhibitors [30]. According to a meta-analysis, the risk of VTE was significantly increased (RR: 1.33 (95% CI: 1.13–1.56); *p* < 0.001) in bevacizumab-treated patients compared to controls, and rates of any-grade VTE were as high as 11.9% with bevacizumab treatment [15]. In comparison, in our cohort VTE developed in only seven (2.8%) patients, which refers to a cumulative incidence rate for all-grade VTE of 2.4% at 12 months.

According to the literature, bleeding of any grade may occur in up to 15% of patients treated with sorafenib [18] with up to 9% experiencing serious hemorrhagic events [31]. However, bleeding risk might be underestimated, as patients with advanced liver function impairment are regularly excluded from clinical trials [19,32]. In our study, 55% of patients had Child–Pugh stage B and C cirrhosis. Nevertheless, bleeding rates were only slightly above what has been observed in phase III trials with 19% of patients experiencing a bleeding episode in our cohort. Importantly, bleeding rates were comparable between patients with different severity of liver disease. Notably, results of the IMBRAVE-150 study showed that the risk of upper gastrointestinal hemorrhage was higher for atezolizumab plus bevacizumab when compared to sorafenib (7% vs. 4.5%) [7]. Therefore, sorafenib might be a safer treatment option in patients with a high bleeding risk (e.g., in patients with advanced liver cirrhosis and a history of portal hypertensive bleeding).

We have to acknowledge some limitations for this study. First, due to the retrospective design of the study we might have missed some events due to a lack of documentation. However, we thoroughly screened all outpatient documentation as well as discharge letters—therefore, we are confident that we can report at least all events that led to medical contact. Additionally, due to the retrospective design, information on all relevant cardiovascular risk factors was not available in every patient and, therefore, we had to exclude a substantial number of patients from calculation of Framingham risk score. Moreover, we observed that patients with more severe disease were checked in more detail; therefore, we might have an overrepresentation of sicker patients in our cohort of patients with available cardiovascular risk evaluation.

## 4. Materials and Methods

### 4.1. Study Design

We retrospectively included patients treated with sorafenib for advanced HCC between 05/2006 and 03/2020 at the Division of Gastroenterology and Hepatology, Vienna General Hospital/Medical University of Vienna. Diagnosis of HCC was established either by histology or dynamic imaging (computed tomography [CT]/magnetic resonance imaging [MRI] scans) according to the European Association for the Study of the Liver (EASL) guidelines [2]. Retrospective data analysis was approved by the local ethics committee of the Medical University of Vienna (1759/2015 and 2033/2017).

### 4.2. Patients and Definitions

Eligible patients were adult (>18 years), diagnosed with HCC, and treated with sorafenib. Patients receiving combination treatments (e.g., with local ablative therapy/chemoembolization/SIRT) and patients with insufficient records were excluded from this study. Patient characteristics and information on family history and metabolic risk factors (e.g., diabetes mellitus), laboratory parameters, tumor characteristics, and Eastern Cooperative Oncology Group performance status (ECOG PS) were collected from the clinical documentation system. MELD and Child–Pugh scores were used to assess liver function at baseline. Obesity was defined as according to current WHO guidelines as a BMI ≥ 30 kg/m^2^ [33].

### 4.3. Determination of Cardiovascular Risk—Framingham Risk Score

In the general population, risk of developing cardiovascular disease (CVD) can be estimated by calculating scores, such as the Framingham risk score [34]. This well validated score includes the parameters age, sex, smoking status, total blood cholesterol, high density lipoprotein (HDL) cholesterol, blood pressure, use of antihypertensive medication, and presence of diabetes mellitus. The output of the score is a point value between −4 to +36 points, which directly translates to the risk of the patient to develop a cardiovascular event within the next 10 years. In order to facilitate interpretation, three risk categories were defined. A risk according to Framingham score <10% at 10 years is considered low, 10–20% confers an intermediate, and >20% a high risk for developing a CVD event [35].

### 4.4. Statistics

Statistical analyses were performed using IBM SPSS Statistics 26 (SPSS Inc., Armonk, NY, USA), R 3.4.1 (R Core Team, R Foundation for Statistical Computing, Vienna, Austria), and GraphPad Prism 8 (GraphPad Software, La Jolla, CA, USA). Continuous variables were reported as mean ± standard deviation (SD) or median (range), and categorical variables were shown as numbers (*n*) and proportions (%) of patients. Comparisons of proportions and of continuous variables were performed by chi-squared test and Student’s *t*-test, respectively. Overall survival (OS) was defined as the time from sorafenib start until date of death or last contact. Time to progression (TTP) was defined as time from sorafenib start to first radiological progression. Patients without progression were censored at the date of last imaging. Only patients with at least one follow-up imaging were included in TTP analyses. Moreover, incidence rates were assessed using competing risk analysis, considering death as competing risk. Therefore, Fine and Gray competing risks regression models (cmprsk: subdistribution analysis of competing risks, https://CRAN.R-project.org/package = cmprsk) [36] were calculated. Multivariable Cox regression analysis with backward elimination was used for evaluation of prognostic parameters independently associated with survival. Survival curves were calculated using the Kaplan–Meier method and compared by log-rank test. A two-sided *p*-value ≤ 0.05 was considered statistically significant.

## 5. Conclusions

In summary, we provide real-world evidence that sorafenib represents a safe treatment option in patients with cardiovascular risk factors, including those with stable coronary heart disease and arterial occlusive disease. The incidence of cardiovascular complications was low, even in those with a high cardiovascular risk profile according to Framingham risk score. Bleeding complications during sorafenib treatment were frequent but within the range of previous reports, even though we also included patients with more advanced liver disease.

## Figures and Tables

**Table 1:** Patient characteristics.

Patients	Characteristics/Statistics	Number (%) or Mean ± SD/Median (Range)
	N, %	252 (100%)
Age (years)	Mean ± SD	66 ± 9.4
	Range	28–88
Sex	Male	215 (85%)
	Female	37 (15%)
Etiology	ALD	93 (37%)
	Viral	71 (28%)
	Unknown	53 (21%)
	Other	35 (14%)
Cirrhosis ^a^	No Yes	55 (22%) 188 (75%)
Child–Pugh Classification	A	114 (45%)
	B	95 (38%)
	C	43 (17%)
ECOG PS	0	149 (59%)
	≥1	103 (41%)
Macrovascular	No	128 (51%)
Invasion	Yes	124 (49%)
Extrahepatic	No	176 (70%)
Metastases	Yes	76 (30%)
BCLC stage	A	18 (7%)
	B	45 (18%)
	C	146 (58%)
	D	43 (17%)
Prior therapy	None	141 (56%)
	TACE/MWA/RFA	59 (23%)
	Resection	12 (5%)
	Other	40 (16%)
AFP (IU/mL) ^b^	Median (Range)	49 (1–50,000)
Platelet Count (G/L) ^c^	Median (Range)	148 (29–778)
Diabetes	NIDDM	76 (30%)
	IDDM	19 (8%)
	None	157 (62%)
BMI (kg/m^2^) ^d^	Mean ± SD	26 ± 5.7
Obesity (BMI ≥ 30 kg/m^2^)		58 (23%)
Arterial Hypertension ^e^	No	96 (38%)
	Yes	154 (62%)
Antihypertensive treatment *	No	140 (56%)
	Yes	112 (44%)
Smoking	No	146 (58%)
	Yes	106 (42%)
Hypercholesterinemia ^f^ (cutoff > 200 mg/dL)	No	177 (76%)
	Yes	55 (24%)
Statin therapy	No	214 (85%)
	Yes	38 (15%)
Anticoagulation ^+^	No	214 (85%)
	Yes	37 (15%)
Antiplatelet therapy	No	211 (84%)
	Yes	37 (15%)
Hypertriglyceridemia ^g^ (cutoff > 200 mg/dL)	No	216 (93%)
	Yes	16 (7%)
Coronary heart disease	No	229 (91%)
	Yes	23 (9%)
Arterial occlusive disease	No	230 (92%)
	Yes	22 (8%)
Renal function according to CKD stages (GFR in mL/min)	1	196 (78%)
	2	38 (15%)
	3	16 (6%)
	4	1 (0.4%)
	5	1 (0.4%)

Missing values: ^a^ *n* = 243 patients, ^b^ *n* = 221 patients, ^c^ *n* = 240 patients, ^d^ *n* = 247 patients, ^e^ *n* = 250 patients, ^f^ *n* = 232 patients, ^g^ *n* = 232 patients. * Excluding propranolol, carvedilol, furosemide, and aldactone. ^+^ Anticoagulation refers to the use of low molecular weight heparin (LMWH), phenprocoumon, and direct oral anticoagulants. Abbreviations: AFP, α-fetoprotein; ALD, alcoholic liver disease; BCLC, Barcelona clinic liver cancer; BMI, Body Mass Index; CKD, chronic kidney disease; ECOG PS, Eastern Cooperative Oncology Group performance status; GFR, glomerular filtration rate; IDDM, insulin-dependent diabetes mellitus; MWA, microwave ablation; NIDDM, non-insulin dependent diabetes mellitus; RFA, radiofrequency ablation; TACE, transarterial chemoembolization.

**Table 2:** Summary of patients with arterial and venous thromboembolic complications.

	Patient 1	Patient 2	Patient 3	Patient 4	Patient 5	Patient 6	Patient 7	Patient 8
Event	MCI/PVT	Ischemic stroke	DVT/PVT	DVT/PVT	DVT	PE	DVT	PE
CPS	B	A	A	A	A	A	B	A
BCLC	C	C	C	C	C	A	C	C
Etiology	Unknown	Viral	ALD	ALD	Unknown	Unknown	ALD	Unknown
Macrovascular Invasion	Yes	Yes	No	Yes	Yes	No	Yes	Yes
Extrahepatic Metastases	No	No	Yes	Yes	Yes	No	No	No
AFP (IU/mL)	3.8	2.5	31.8	-	866.0	-	-	17,312.0
Known coronary heart disease	No	No	No	No	No	No	No	No
Known arterial occlusive disease	No	Yes	No	Yes	No	No	No	No
Diabetes mellitus	No	Yes	No	No	Yes	Yes	Yes	No
Arterial hypertension	No	Yes	No	No	No	Yes	No	No
Framingham risk score points (risk, %)	11 (7.3%)	17 (29.4%)	N/A	N/A	N/A	N/A	N/A	N/A
Framingham risk class	Low	High	Low	High	High	N/A	High	N/A
Time to (first) event (months)	1.4	19.6	15.9	3.8	3.8	3.4	2.7	11.0
Sorafenib Dose at event (mg)	800	200	800	800	400	800	800	400
Event Management	Sorafenib: Discontinued	Sorafenib: Interrupted	Sorafenib: Continued				Sorafenib: Continued	Sorafenib: Continued
	Medical treatment: DAPT (ASA + Clopidogrel)	Medical treatment: none	Medical treatment: anti-coagulation (LMWH)	N/A	N/A	N/A	Medical treatment: anti-coagulation (LMWH)	Medical treatment: anti-coagulation (LMWH)
	Intervention: PCI	Intervention: none	Intervention: none				Intervention: none	Intervention: none
Death within 14 days (yes/no)	Yes	No	No	No	Yes	No	No	No

Abbreviations: AFP, α-fetoprotein; ALD, alcoholic liver disease; AOD, arterial occlusive disease; ASA, acetylsalicylic acid; BCLC, Barcelona Clinic Liver Cancer; CPS, Child–Pugh Score; DAPT, dual anti-platelet therapy; DM, diabetes mellitus; DVT, deep vein thrombosis; LMWH, low molecular weight heparin; MCI, myocardial infarction, N/A, not available; PE, pulmonary embolism; PVT, portal vein thrombosis.

**Table 3:** Multivariable analysis of prognostic factors.

Patient Characteristics		HR	95% CI	*p*-Value (Cox Regression)
Cardiovascular risk (Framingham risk score)	Low/intermediate	1		
	High	1.53	1.07–2.19	0.019
BCLC stage	A	1		
	B	1.04	0.48–2.24	0.928
	C	1.21	0.63–2.30	0.568
	D	2.34	1.16–4.71	0.018
AFP (IU/mL)	≤400	1		
	>400	2.04	1.44–2.90	<0.001

Abbreviations: AFP, α-fetoprotein; BCLC, Barcelona Clinic Liver Cancer; HR, Hazard Ratio; 95% CI, 95% confidence interval.

**Table 4:** Bleeding risk according to Child–Pugh stage.

Type of Bleeding Event	CPS A (*n* = 114)	CPS B (*n* = 95)	CPS C (*n* = 43)	*p*–Value
Gastrointestinal bleeding	13 (11.4%)	13 (13.7%)	9 (21%)	0.305
Epistaxis	5 (4.4%)	6 (6.3%)	1 (2.3%)	0.576
Intracerebral hemorrhage	-	1 (1%)	-	-
Hemorrhoidal hemorrhage	2 (1.8%)	-	1 (2.3%)	0.382
Gingival bleeding	1 (0.9%)	-	-	-
Any bleeding	20 (17.5%)	18 (18.9%)	10 (23.3%)	0.718

Abbreviations: CPS, Child–Pugh stage.

## Supplementary Materials

The following are available online at https://www.mdpi.com/2072-6694/12/10/2961/s1, Figure S1: Competing risk analysis comparing the cumulative incidence of thromboembolic events in Child-Pugh A vs. B/C patients, Figure S2: Time to progression (TTP) of patients with low/intermediate risk vs. high risk according to Framingham score, Figure S3: Competing risk analysis of the development of vascular events (thromboembolic and bleeding) with respect to sorafenib starting dose, considering death as competing risk, Table S1: Comparison of baseline characteristics between patients who did versus did not experience thromboembolic complications during sorafenib treatment, Table S2: Univariate analysis of prognostic factors for overall survival in patients with HCC treated with sorafenib, Table S3: Association of baseline characteristics with the occurrence of bleeding events.
