# Supplementary material for: Vascular Complications in Patients with Hepatocellular Carcinoma Treated with Sorafenib
Source: Cancers (Basel). 2020 Oct 13;12(10):2961. doi: 10.3390/cancers12102961 (PMC7602103; doi:10.3390/cancers12102961)
Supplement: Supplementary file 1 [file cancers-12-02961-s001.pdf]

cancers

MDPI

Supplementary Material

Vascular Complications in Patients with Hepatocellular Carcinoma Treated with Sorafenib

Katharina Pomej, Bernhard Scheiner, Dabin Park, David Bauer, Lorenz Balcar, Tobias Meischl, Mattias Mandorfer, Thomas Reiberger, Christian Müller, Michael Trauner and Matthias Pinter

Table S1. Comparison of baseline characteristics between patients who did versus did not experience thromboembolic complications during sorafenib treatment.

		Without thromboembolic complication (n=244)	With thromboembolic complication (n=8)	p-value
		Number (%) or mean±SD / median (range)		
**Age (years)**		65±9.5	66±7.7	**0.748**
**Sex**	Male	209 (86%)	6 (75%)	**0.333**
	Female	35 (14%)	2 (25%)	
**Etiology**	ALD	90 (37%)	3 (38%)	**0.160**
	Viral	70 (29%)	1 (12%)	
	Unknown	49 (20%)	4 (50%)	
	Other	35 (14%)	-	
**Cirrhosis**	Yes	182 (75%)	6 (75%)	**1.000**
**Child-Pugh Classification**	A	108 (44%)	6 (75%)	**0.184**
	B	93 (38%)	2 (25%)	
	C	43 (18%)		
**ECOG PS**	0	144 (59%)	5 (63%)	**0.898**
	≥1	100 (41%)	3 (37%)	
**Macrovascular invasion**	Yes	118 (48%)	6 (75%)	**0.172**
**Extrahepatic Metastases**	Yes	73 (30%)	3 (38%)	**0.702**
**BCLC stage**	A	17 (7%)	1 (13%)	**0.216**
	B	47 (19%)	-	
	C	139 (57%)	7 (87%)	
	D	41 (17%)	-	
**Prior therapy**	None	137 (56%)	4 (50%)	**0.856**
	TACE/MWA/RFA	57 (23%)	2 (25%)	
	Resection	11 (5%)	1 (13%)	
	Other	39 (16%)	1 (12%)	
**AFP (IU/mL)**	Median (range)	50 (0.8-50000)	32 (32-2.5)	**0.840**
**Platelet Count (G/L)**	Median (range)	146 (29-778)	237 (115-425)	**0.076**
**Diabetes**	Yes	91 (37%)	4 (50%)	**0.480**
	No	153 (63%)	4 (50%)	
**BMI (kg/m2)**	Mean±SD	26±5.7	26±4.7	**0.908**
**Obesity (BMI ≥ 30kg/m2)**	Yes	56 (23%)	2 (25%)	**1.000**
**Arterial Hypertension**	Yes	152 (62%)	2 (25%)	**0.057**
**Antihypertensive treatment**	Yes	111 (45%)	1 (12%)	**0.079**
**Smoking**	Yes	100 (41%)	6 (75%)	**0.072**
**Hypercholesterinemia (cut-off >200mg/dL)**	Yes	53 (22%)	2 (25%)	**1.000**
**Statin therapy**	Yes	36 (15%)	2 (25%)	**0.346**
**Hypertriglyceridemia (cut-off >200mg/dL)**	Yes	14 (6%)	2 (25%)	**0.098**

**Coronary heart disease**	Yes	23 (9%)	-	**1.000**
**Arterial occlusive disease**	Yes	20 (8%)	2 (25%)	**0.148**

Abbreviations: AFP, α-fetoprotein; ALD, alcoholic liver disease; BCLC, Barcelona clinic liver cancer; BMI, Body Mass Index; ECOG PS, Eastern Cooperative Oncology Group performance status; MWA, microwave ablation; RFA, radiofrequency ablation; TACE, transarterial chemoembolization;.

Table S2. Univariate analysis of prognostic factors for overall survival in patients with HCC treated with sorafenib.

		**Overall survival (months)**			**p-value**
		**N**	**Median**	**95% CI**	**(log rank)**
**Age**	< 65 years	112	10.9	8.6-13.3	**0.515**
	≥ 65 years	140	8.9	7.1-10.6	
**Gender**	Male	215	10.2	8.4-12.1	**0.237**
	Female	37	7.2	4.4-10.1	
**Child-Pugh Classification**	A	114	13.0	10.5-15.3	**0.000**
	B	95	7.8	6.0-9.6	
	C	43	4.5	1.2-7.8	
**Cardiovascular risk (Framingham risk score)**	Low/intermediate	119	10.0	6.8-13.2	**0.055**
	High	54	7.4	3.4-11.3	
**Ascites**	No	146	12.5	9.9-15.1	**0.000**
	Yes	106	6.0	3.7-8.4	
**ECOG PS**	0	149	11.5	8.0-15.1	**0.000**
	≥ 1	103	6.6	4.0-9.1	
**Macrovascular Invasion**	No	125	12.3	9.9-14.7	**0.002**
	Yes	124	7.3	5.5-9.1	
**Extrahepatic spread**	No	174	10.0	8.3-11.7	**0.722**
	Yes	76	7.9	3.5-12.3	
**BCLC stage**	A	18	13.0	8.6-17.3	**0.000**
	B	47	12.0	9.9-14.4	
	C	146	9.4	7.4-11.5	
	D	41	4.5	1.3-7.8	
**AFP (IU/mL)**	≤200	134	10.9	9.1-12.7	**0.001**
	>200	87	5.4	2.3-8.5	

Abbreviations: AFP, α-fetoprotein; BCLC, Barcelona clinic liver cancer; ECOG PS, Eastern Cooperative Oncology Group performance status.

Table S3. Association of baseline characteristics with the occurrence of bleeding events.

		**Without bleeding complication (n=204)**	**With bleeding complication (n=48)**	**p-value**
		**Number (%) or mean±SD / median (range)**		
**Age (years)**		65±9.6	66±8.5	**0.875**
**Sex**	Male	172 (84%)	43 (90%)	**0.353**
	Female	32 (16%)	5 (10%)	
**Etiology**	ALD	76 (37%)	17 (35%)	**0.764**
	Viral	55 (27%)	16 (33%)	
	Unknown	45 (22%)	8 (17%)	
	Other	28 (14%)	7 (15%)	
**Cirrhosis**	Yes	148 (73%)	40 (83%)	**0.270**
**Child-Pugh Classification**	A	94 (46%)	20 (42%)	**0.718**
	B	77 (38%)	18 (37%)	
	C	33 (16%)	10 (21%)	
**ECOG PS**	0	120 (59%)	29 (60%)	**0.973**

	≥1	84 (41%)	19 (40%)	
**Macrovascular invasion**	Yes	101 (50%)	23 (48%)	**0.772**
**Extrahepatic Metastases**	Yes	63 (31%)	13 (27%)	**0.578**
**BCLC stage**	A	13 (6%)	5 (10%)	**0.547**
	B	39 (19%)	8 (17%)	
	C	121 (59%)	25 (52%)	
	D	31 (15%)	10 (21%)	
**Prior therapy**	None	117 (57%)	24 (50%)	**0.347**
	TACE/MWA/RFA	46 (23%)	13 (27%)	
	Resection	9 (4%)	3 (6%)	
	Other	32 (16%)	8 (17%)	
**AFP (IU/mL)**	Median (range)	45 (0.8-50000)	108 (1-50000)	**0.503**
**Platelet Count (G/L)**	Median (range)	145 (29-778)	154 (47-420)	**0.942**
**Arterial Hypertension**	Yes	123 (60%)	31 (65%)	**0.636**
**Antihypertensive treatment**	Yes	90 (44%)	22 (46%)	**0.830**
**Smoking**	Yes	84 (41%)	22 (46%)	**0.557**
**Coronary heart disease**	Yes	20 (10%)	3 (6%)	**0.583**
**Arterial occlusive disease**	Yes	19 (9%)	3 (6%)	**0.776**
**Anticoagulation**	Yes	27 (13%)	10 (21%)	**0.186**
**Antiplatelet therapy**	Yes	33 (16%)	4 (8%)	**0.404**

Abbreviations: AFP, α-fetoprotein; ALD, alcoholic liver disease; BCLC, Barcelona clinic liver cancer; BMI, Body Mass Index; ECOG PS, Eastern Cooperative Oncology Group performance status; MWA, microwave ablation; RFA, radiofrequency ablation; TACE, transarterial chemoembolization;.

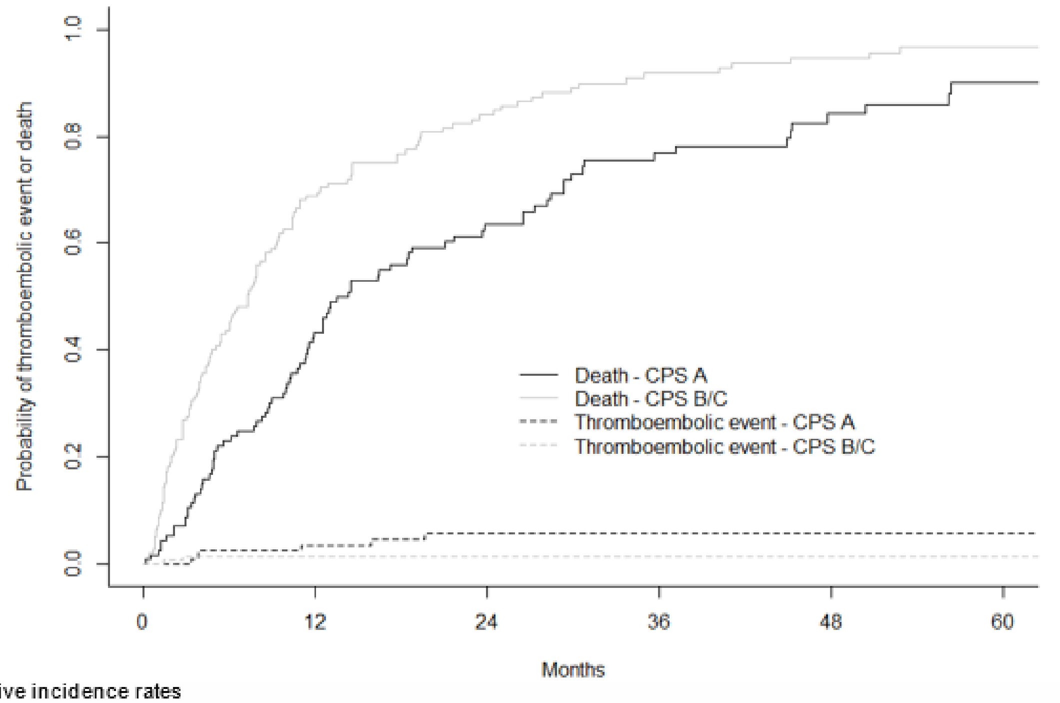

Cumulative incidence rates					
Death CPS A	43.3%	63.6%	76.8%	84.2%	90.1%
Death CPS B/C	68.8%	84.0%	91.8%	94.7%	96.6%
Thromboembolic event CPS A	3.6%	5.7%	5.7%	5.7%	5.7%
Thromboembolic event CPS B/C	1.5%	1.5%	1.5%	1.5%	1.5%

Figure S1. Competing risk analysis comparing the cumulative incidence of thromboembolic events in Child-Pugh A vs. B/C patients. Abbreviations: CPS, Child-Pugh-Score

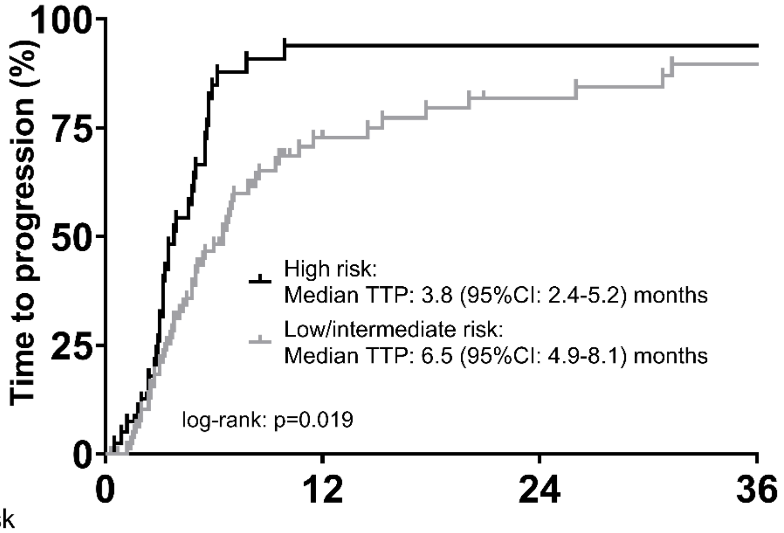

No. at risk				
High	40	3	8	5
Low/intermed.	81	14	3	3

Months

Figure S2. Time to progression (TTP) of patients with low/intermediate risk vs. high risk according to Framingham score. Abbreviations: TTP, time to progression

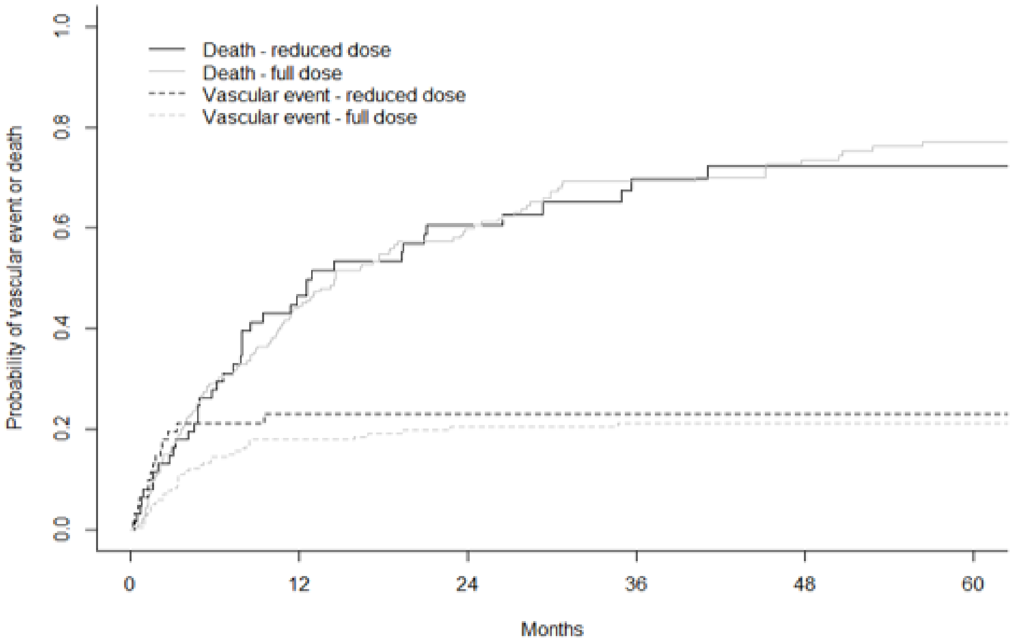

Cumulative incidence rates					
Death reduced dose	46.4%	60.6%	69.8%	72.2%	72.2%
Death full dose	44.5%	59.8%	69.3%	73.5%	77.0%
Vascular event reduced dose	23.0%	23.0%	23.0%	23.0%	23.0%
Vascular event full dose	17.9%	20.5%	21.2%	21.2%	21.2%

Figure S3. Competing risk analysis of the development of vascular events (thromboembolic and bleeding) with respect to sorafenib starting dose, considering death as competing risk.

© 2020 by the authors. Licensee MDPI, Basel, Switzerland. This article is an open access article distributed under the terms and conditions of the Creative Commons Attribution (CC BY) license (http://creativecommons.org/licenses/by/4.0/).
